# European Correspondence

**Published:** 1868-01

**Authors:** 


### EUROPEAN CORRESPONDENCE.

PARIS, FRANCE, NOV. 23, 1867.

*To Editors Atlanta Medical and Surgical Journal:*

It was announced that, on the 4th instant, the course of lectures at the School of Medicine would commence, and accordingly, on the 5th I went to hear Robin, the great French histologist The amphitheatre was densely crowded with students and physicians, many of whom were doubtless anxious to hear his lecture. We were disappointed, however, for as soon as the Professor entered and took his stand, a number of the students commenced to sing, hiss and imitate all manner of animals, so that if one had to judge of his whereabouts by the sense of hearing alone, he would have supposed himself in a menagerie, rather than in an institution where science is taught. After several unsuccessful attempts to commence, there was no alternative but to leave. I learned, for I did not go again, that this state of things continued for at least a week. The trouble, it seems, was a dissatisfaction with some changes made by the President, whom they desired to deliver, as heretofore, the introductory address, in order that they might have an opportunity to manifest their displeasure.

During the past year there have been several deaths and one resignation among the members of the Faculty, in consequence of which some new men have been elected. Nélaton lectures no more. He has given up his clinic. He seems to be surfeited with clinics and retires loaded down with honors, received from his country and the Emperor. Why is this? Rumor says that he will enter politics—that more honors than have already been bestowed are to be heaped upon him by the Emperor. Be this as it may, for the future his valuable services are lost to the profession.

His resignation and the death of Velpeau have within a few months deprived the profession here of its two greatest surgeons—who have not only stood at the head of their profession in Paris for more than a quarter of a century, but who have by their labors and genius won an enviable reputation in every country. Prof. Jarjavy conducts the clinics of Nélaton at the Clinique de Faculté, and Prof. Gosslin that of Velpeau at the Charité.

I was present at the latter to hear the opening lecture of Prof. Gosslin, who referred touchingly to the memory of Velpeau in these words: "When I took possession," said he, "of the service of surgery in this hospital, I have had to be affected by all the remembrances that I met here. To-day, in speaking in this amphitheatre, I am still more so in thinking of Prof. Valpeau, who preceded me. It seems to me that I am still listening to the remarkable lessons of this great master, of whom I had the good fortune of being a pupil from 1840 to 1843. Velpeau was then in the fullness of his talent, which has known no decline. I was not a beginner, but was at a period of my studies where I could understand and appreciate him. Indeed, I had some tendency to official teaching, but I was far from expecting the perilous honor of succeeding him in the future. I then place myself under the patronage of Velpeau; I will make efforts to imitate him. If it is not given to me to have the same strength of judgment and memory, I hope I shall have the same regularity, the same punctuality, the same consciencious discharge of duty, the same love for the patient, and the same zeal in teaching; and if I ever fail, the examples left by Velpeau will support and encourage me in the mission I have to fulfill."

I happened, very opportunely, in the lying-in-room of Prof. Dupal, about two weeks since, while there were four cases in labor. Among them was one of more than ordinary interest. She had already been in labor more than forty hours. On the 6th instant, at midnight, she was brought in from the ward; at 11 A. M. on the 7th the membranes were ruptured. The pains continued good up to 1½ P. M. on the 8th, when it was found upon ausculation that the action of the fœtal heart was becoming more and more feeble. The condition of the woman was good, though she was very much fatigued from the protracted labor. A further examination, per vaginum, revealed the os uteri insufficiently dilated to admit of delivery. There were no cicatrices to be found there, nor did there seem to be any interstitial deposit, but the insufficient dilatation appeared to be due entirely to the loss of the bag of waters—the natural dilator of the os.

Under these circumstances, two courses presented themselves, viz: 1st, As the dilatation of the os was insufficient to admit of delivery, to let the labor take its natural course, and hazard the life of the child. 2d, To interfere and endeavor to save the child.

The latter, being clearly preferable, was adopted, and it was determined to deliver at once with the forceps. In order to apply them, an incision was made in the neck of the uterus on both sides with a blunt pointed bistoury. Then the presentation being transverse, with the face to the right, the forceps were applied and the delivery effected at once, the face passing to the front under the arch of the pubis. The child was asphyxiated when born, but respiration was soon established by the ordinary means. The mother has entirely recovered. This favorable result to both mother and child proves the wisdom of the course pursued.

In the surgical ward of the Clinique de la Faculté there is at this time a very interesting case of popliteal aneurism, in the left leg of a man aged thirty-seven years. It has existed a long time, and reached a size considerably larger than his two fists. Prof. Jarjavy, in whose service he is, treated it by digital compression of the femoral artery above, in order to produce coagulation of the blood in the sack, and thereby cure the aneurism. Compression was kept up for twelve hours, when all pulsation ceased. In four days it returned; compression was used again for six hours. In four days after this last the pulsation returned again; the compression was reapplied for four hours, which has resulted in the obliteration of the sack.

But the most interesting feature in connection with this case is, that in a short time after the last compression, gangrene of the integument surrounding the aneurismal tumor took place at two points, exposing to view the large clot in the sack. This result is both interesting and instructive, and should not be lost sight of as one of the possible results of this method of treatment.

Before this result the collateral circulation, in the superior and inferior, external and internal articular arteries of the popliteal space, was distinctly felt and well established. Nor has the limb below the knee suffered in the least. The sloughing of the integument then, it seems, can only be attributed to the pressure exerted by the clot from within, preventing the free circulation of a supply of blood already deficient in quantity.

A section of the clot presents a fine view of the successive strata of fibrin which have been deposited, their different thicknesses, the ease with which they separate, their elacticity, &c. This tumor is diminishing in volume daily, under the process of suppuration, and thereby increasing the danger of secondary hemorrhage. In my next letter I will give you the result of the case.

W. S. Armstrong, M. D.

PARIS, OCT. 2, 1867.

DEAR DOCTOR—Though I shall always look back with satisfaction upon the months I spent in London, I find Paris affords incomparably greater opportunities for the study of those affections in which I am at present particularly interested. The St. Eugénie and the Hospital Des Enfants Malades, containing between them nearly a thousand beds, are exclusively children's hospitals, and in these institutions the amplest advantages for observing all infantile diseases are afforded. The Hospital Du Midi, of 340 beds, is devoted entirely to venereal diseases, and the St. Louis, with 700 beds, contains at all times almost every example of cutaneous affections. In the latter, venereal cases are also admitted. Besides these special hospitals, there are eleven others for general diseases, making in all fifteen hospitals, with an aggregate of nearly twenty thousand beds. As you are aware, no distinction of color is observed in France, and during my first visit to the St. Louis I observed a fine looking mulatto man amongst Mr. Hardy's students, and in one of the wards I saw by the side of other patients a negro from Cuba. In this hospital there are at present two cases of leprosy, both in young men, and one of them from New Orleans. Another case of this disease, in a girl, has just been sent to the hospital for incurables. In all these cases the disease was of tropical origin. There is also in the St. Louis a splendid case of Elephantiasis Arabica, or Barbadoes leg, often erroneously called leprosy. The patient appeared to be in tolerable health, exhibiting, in fact, no signs of disorder, except in the lower extremeties and scrotum. The former are nearly as large as a man's body, and almost as hard as wood. His scrotum is the size of a man's head, indurated, and covered with wart-like excrescences. A man of twenty-one or twenty-two years, without testicles and only the merest trace of a scrotum, and a penis the size of a lead-pencil and only two inches long, presents an interesting case of arrest of development. My attention was called to a woman in whom the left femur was nearly one-half shorter than the right, and who, notwithstanding, walked without perceptible limping. Being the subject of a venereal affection, she was placed on a couch for examination, when, her extremeties being exposed, the deformity was discovered. The limb had been badly fractured in her childhood, and from some cause the ends of the bone had been allowed to overlap and induce the shortening of the limb spoken of. The poor woman concealed her lameness by walking with the sound leg immensely bent, impelled, as was suggested by the surgeon in attendance, by her true femenine instincts to hide her deformity from the sex upon whose fancy she depended for subsistence. A case of chronic *eczema rubrum*, in which the cutaneous affection alternated with a most violent and dangerous bronchitis, was shown me. The subject was a man beyond middle life, and the complaint involved both the trunk and extremeties. The eczema yielded somewhat readily to treatment, but no sooner was the skin disease subdued than the pulmonary trouble came on, and when that is relieved, out crops the eczema again.

The treatment for itch and the remedy for favus at the St. Louis might be classed with the practice styled "heroic," if there was anything of heroism in cramming drugs down a sick man's throat or torturing him by harsh operations. The treatment in favus consists chiefly in *épilation*—the extraction of all the diseased hairs by means of forceps, repeating the operation time and again; for, unfortunately, the first, or second, or third, is not always rewarded with success. When a large portion of the scalp is affected by favus, and tender as it always is, you may imagine what the sufferings of a patient must be, subjected to épilation. Prior to extracting the hairs, the scalp is rubbed with juniper oil, which is said to render the operation less painful. The after-treatment consists in daily applications of turbith ointment, with attention, of course, to the general health of the patient. Another plan of treatment, called the "Mahon treatment," to apply to the scalp, from which the hair has been closely cut and the scabs carefully removed by poultices and bathing, the following ointment:

℞—Lard, grammes, 80.
Soda of Commerce, grammes, 15.
Slacked Lime, grammes, 10.

Mix carefully.

The itch victims are treated as follows: At one o'clock the patients are called in the large bath-room of the hospital, and sometimes number fifty subjects. Here they are required to strip, and being furnished with a supply of soft soap, they proceed to rub themselves with it thoroughly, assisting each other mutually in the process. Having been well anointed, they repair to warm baths, where they wash off the soap. They are then provided with sulphuret of lime ointment, with which they anoint themselves minutely and carefully, and afterwards wash off in another warm bath, when they are pronounced cured. The operation lasts about two hours. Many of the poor fellows are nearly raw from scratching when they enter the hospital, and others being covered with scabs, which must be removed by the detergent process, it is easy to comprehend that the treatment is not unattended by pain, and, unfortunately, it is not infallible. For a public institution where itch patients resort in great numbers, it is probably the best plan of treatment. It is economical, saves time, and is comparatively certain; but in private practice I should always greatly prefer the simple warm bath and castile soap, followed afterwards by sulphur ointment.

A singular example of the influence of the imagination upon the physical system occurred in this hospital a few days ago. One of the patients laboring under elephantiasis had a hemorrhage from the nose which resisted all the usual remedies and was becoming alarming. In fact, his life seemed to be in imminent danger. Plugging the nose was resolved upon, the nature of the process was explained to him, and the sponge, cat gut, etc., exhibited. The young man was so much alarmed at the thought of having to submit to so formidable a procedure, that the epistaxis ceased at once, and after three days has not returned.

An operation intended to supersede paracentisis was performed in one of the hospitals here a few days since. A patient laboring under general dropsy, in which the encroachment of the fluid upon the lungs was causing embarrassed respiration, was punctured in fifty or sixty places upon the abdomen and legs, with temporary relief. The punctures were made with an exploring needle, and only extended through the skin. Serum began at once to exude, and continued to flow for many hours, during which the subsidence of the edema of the face, limbs, and scrotum, was most striking, and with it the breathing became easy. The patient seemed not to suffer from the operation.

The physicians here are using, with much eclat, a new remedy in the treatment of ulcers, chancres, and such cases —*iodoform.* It is a yellowish powder, consisting of small shining crystals, resembling those of sulphur. Its odor is that of iodine, and is so persistent, that, having taken some of it into my fingers at 9 o'clock in the morning, it was disagreeably perceptible at bed time that night, notwithstanding repeated ablutions. It is claimed for this remedy that while it possesses all the curative powers of iodine, it is painless in its action—in truth is an anesthetic. Wonderful accounts of its efficacy are related, but I am not able to speak of its merits from any personal observation. Like chloroform, which lay long idle after its discovery, iodoform, though long known to chemists, is now for the first time attracting attention as a curative agent. It would be singular if it should attain the distinction to which its confrere has risen.

I have been gratified to find that *specialism* is as thoroughly discountenanced by the profession in this great metropolis as it is in London, and that a medical man who proclaims himself, through the press or by private cards, a candidate for practice in any exclusive class of diseeses, places himself beyond the pale of regular medicine. Not that devotion to special branches of the art is discountenanced, for in this way it is now acknowledged the greatest advances in medicine are made; bnt the candidate for public favor must pursue the legitimate channels of the profession. If he writes good books, like Ricord, and proves himself a master of his subject, the profession will honor him as their leader. Our countryman, Dr. Marion Sims, has won an enviable distinction by his writings and by his skill in uterine surgery, and is reaping an ample harvest of fame and fees. Mr. Ereomus Wilson, Dr. Tilbury Fox, and Mr. Milton, of London, are all distinguishing themselves in the line of cutaneous diseases, and all in a way to command the respect and admiration of their brethren. But advertising doctors are repudiated and scouted. They are assigned to the same category with "Indian Doctors," "Cancer Doctors," and dealers in clairvoyance and spiritualism.

Yours truly, L. P. Y., Jr.

---

PARIS, RUE JACOB, OCT. 10, 1867.

MY DEAR DOCTOR—For nearly two months I have done no work in the field of medicine, but have been pleasantly idling about in flat Holland, and strange Germany, and grand Switzerland, and famous Belgium, and beautiful France; seeing the many sights and wonders of art and nature with which this part of the old world abounds. I have just returned to Paris, and have not yet had time to get to work in the hospitals sufficiently to be able to send you anything of professional interest, but I fear if I postpone much longer the pleasant task of communicating with you, I shall be suspected of having lost sight of medicine.

While in Switzerland, I had an opportunity of seeing something of Goitres and Cretinism, and possibly my observatians may be of interest to you. The first town where I observed goitres was Martigny, a small village of two thousand inhabitants, situated at the head of the valley of the Rhone. The number of persons affected with goitres, and the number of idiots, dwarfs and deformed people in Martigny, and the country surrounding it, and the villages near by, is really appalling; and it seemed to me that one-fourth of the natives that we encountered had goitre to a greater or less extent. In a little village near Martigny I counted four idiotic children in one group, and a short distance off saw two others vacantly staring from windows. Goitre was apparently more common among women than among men, though exceedingly frequent in the latter. The largest tumors that I observed were larger than a man's head. They were not invariably of regular shape, and were sometimes multiple. Most frequently, however, the tumor was single, hanging down from the centre of the neck, extending equally on either side. In some cases the tumors were lobulated, and in several instances I observed a tumor on either side of the neck as large as my fist. It is probable I did not see the worst cases, as I only came in contact with those who were able to labor, and were, therefore, out of doors. This disease seemed to be confined to the inhabitants of the valleys, who are an exceedingly diminutive and miserable looking race of people, with small legs and diminutive faces, reminding me of the description of *Barney Sniffle* in "Georgia Scenes," who, you remember, "lived upon red clay in the winter time, and blackberries in the summer." They have the complexion of the people from the Red River bottoms, or from the swamps of South

Carolina, or the pond settlements near Louisville. They were chilly-looking people, bilious-looking people, and the language of their bodies expressed as clearly as if written in legible characters that they were the subjects of miasmatic poison; and in a conversation upon the subject with an exceedingly intelligent Englishman, I learned that they suffered terribly from fever and ague, and other forms of malarial disease.

The origin of goitre is still a matter of uncertainty among medical men, and is, I believe, generally ascribed to the water used by the people, or to the impure air of their filthy, overcrowded, and ill-ventilated log cabins. The valleys of the Rhone, and the other rivers of Switzerland, are composed of alluvial material, consisting of debris brought down from the mountains year after year. It is stated that the valley of the Rhone has gained about a mile and a half in length since the time when Julius Cæsar marched about this country. As you are aware, the Rhone empties into Lake Geneva. It is the fiercest little river imaginable, and dashes along like a horse running away. Its waters are almost milky in color, from the particles of granite and other rocks which they carry along with them, and every moment in the day it is tumbling its heaps of debris into the lake. At one period the greater portion of these valleys was little more than useless marshes, ever breeding pestilence, but by a system of drainage, and by walling the rivers in, so as to confine them to narrow beds, these indefatigable little husbandmen have converted the waste and poisonous marshes into arable land, on which my delighted eyes saw fields of Indian corn! With the improvement of the land, a corresponding diminution of disease among the people has occurred. Goitre, cretinism, &c., are less prevalent now than in days gone by, and I am impressed with the belief that this improvement is owing to the draining of the valleys, and that goitre is probably of malarial origin. From the history of the cases of leprosy which I saw in London, and from what I have read on the subject, I am inclined to believe that that disease, too, is of malarial origin. As the cause of neither disease is known, let us say that a vegetable parasite, the product of vegetable decomposition, being taken into the system, is deposited, in one instance, in the thyroid gland, and is developed into a fungus of considerable size; and in the other is deposited in the skin and cellular tissue, causing the tumors, nodules, etc., observed in leprosy. This would be a very satisfactory explanation to at least non-professional readers. I only suggest the idea, and will not attempt to elaborate it; nor am I willing to say such is my settled opinion. The water of both the larger and lesser mountain streams is of a muddy, whitish color, and when held in a glass vessel between you and the sun, you see innumerable particles of granite, etc., glistening in it, and upon drinking some of it, I imagined it made my throat feel gritty. Its long-continued use as a beverage I am sure would produce sand-bars in the stomach, and from never having heard of any such cases, I am compelled to believe these people either filter this water, or else allow it to settle before using it.

Paris is still crowded with people. The weather is raw and cold, and it rains every day. Nélaton has given up teaching and retired from practice, I am told, and it is said the Emperor is going to make him a Peer of France. In the two large hospitals devoted to infantile diseases, and in those devoted to skin diseases and syphilis, I shall find ample work to interest me in Paris as long as I can spare the time to stay, and, as often as possible, I shall drop you a line about Paris physic. Truly yours, L. P. Y., Jr.

---

Paris, November 10th, 1867.

Dear Doctor—You must have remarked how busy death has been in the last few months among the renowned names of our profession. Lawrence, Faraday, Rostan, Trausseau, Velpeau, all have departed in a brief period from the scene of their labors. Faraday, though not a physician, had connected himself to the profession by his applications of electrical science to Medicine, in a way to rank him among the contributors to Medical Science. Foremost among the physicists of his day, his name will go down to future ages in British history, along with those of Sir Isaac Newton and Sir Humphrey Davy. In his hands the science of electricity has received a new momenclature and a new form, and in this field, as well as in the charm of his popular discourses, he has hardly left his equal behind him.

His countryman, Sir William Lawrence, who died on the 15th of July, at the advanced age of eighty-four, was not less distinguished among the Surgeons of his times. Sir William, after spending seven years and a half at a Classical School, was apprenticed to Mr. Abernathy, in whose house he became an inmate, and so impressed was his teacher by the zeal and talents he displayed in his anatomical pursuits that in the third year of his apprenticeship he appointed him demonstrator of anatomy. For twelve years he continued to labor in this position. In 1813 he became assistant Surgeon to St. Bartholomews; and was not full Surgeon till 1824, when he was 41 years of age. In 1815 he was chosen one of the professors of anatomy to the Royal College of Surgeons, at which he delivered the lectures for four years. His connection continued with St. Bartholomew's until nearly the time of his death. For a long period he was also connected with the Eye Infirmary at Moorfields, and was Surgeon to the Royal Hospitals of Bridwell and Bethlehem. Few professional men, in fact, have held office in more public institutions, and rarely has an officer discharged his duties with greater ability. Yet he has not escaped censure, and more than once he was involved in exciting controversies with his colleagues and the officers of hospitals and colleges. On one occasion, when delivering the Hunterian Oration before the College of Surgeons, his line of remarks produced a storm of indignation in his auditory. He has undertaken to defend some unpopular acts of the Council of the College, which he had been heard years before fiercely to denounce. The indignation of those Surgeons who had listened to him then was intense, but the orator was unmoved in the fiercest of the storm, and his eloquence finally triumphed. When he had allowed his audience to exhaust their displeasure, he proceeded to conclude his address in a peroratide which called forth the plaudits of his oratory.

Sir William Lawrence had a more unfortunate controversy with his old friend and preceptor, Mr. Abernathy. In a course of lectures which he delivered before the College of Surgeons on the Natural History of Man, he advocated doctrines not deemed orthodox, and Mr. Abernathy replied to them, attempting to show that they favored materialism. But the blunt, conscientious old Surgeon was no match in eloquence and power for his former pupil. Lawrence triumphed as a controversialist, but was defeated in the end. The discussion attracted public attention to the obnoxious doctrines and he was called upon by the authorities of Bethlehem and Bridwell Hospitals to resign his appointment at those institutions. He did not resign, however, but recanted, and, what is still more damaging to his character, bought up all the copies of his condemned book and sent them over to America. The cordiality between him and Mr. Abernathy was never restored.

It is painful to refer to these short-comings of a man so distinguished for his talents and for his honorable labors as a Surgeon, but it is due to the truth of history that the foibles of his character should be given along with its elevated qualities. No where in the civilized world will any Surgeon hear of the death of Sir William Lawrence without feeling that one of the great lights of Surgery has gone out. Few British Surgeons have written more extensively, and on all the subject of which he has treated he has written ably and well. I make no exception of the unlucky volume which called forth the fierce animadversions of Mr. Abernathy, and I believe after men have had leisure impartially to examine its doctrines they will generally agree in acquitting it of those infidel tendencies of which it was accused when it first appeared. Certainly it is a volume of unusual force of thought and eloquence of style, and places its author among the foremost thinkers of his age.

As an orator, those who have heard Sir William Lawrence, agree that in manner, substance, and expression he had hardly a superior in Great Britain. As a Surgeon he was placed by public opinion at the head of all his predecessors at St. Bartholomews, the illustrious Pott alone excepted. As an operator, though surpassed by Cooper, and it may be by a few others, he ranked amongst the most dextrous of his day. And if in his public life his course sometimes gave color to the charge that "his principles were somewhat lax, his heart was somewhat cold," no one who knew him personally will deny that in all the relations of private life he was most estimable and affectionate. I can never forget the cordial terms in which I have heard his warm hospitality, his blandness and courtiousness of manners, his interest in young strangers, and his kindness to his patients described by our young countrymen who bore letters of introduction to him.

The face of Sir William Lawrance denoted intellectual power. His forehead was high and broad, his mouth large and expressive, his chin massive, indicating firmness of will His eyes were blue, inclining to gray, and suggested the idea of coldness and sagacity. He had a vigorous frame, well-developed, and was in person above the middle height. Early in life he suffered from an attack of facial paralysis, which distorted his features for a time, but he obtained relief from it by abstinence and the loss of blood. Several times afterwards he had paralytic seizures in different limbs, but they yielded without treatment to the force of his excellent constitution. About two years ago his powers of locomotion became seriously impaired, and he was threatened with hemiplegia, but after a time he rallied so far as to resume a portion of his professional duties.] [At last the brain gave way, he became suddenly hemiplegic on the right side, and lost the power of speech. He broke down in the Council Chamber of the College of Surgeons. Despite the affection of the brain which palsied his right side, his splendid intellect remained unimpaired to the last, and though unable to utter his thoughts he manifested a pleasure in the conversation of his family and the friends admitted to his bed-side until within a few hours of his death.

Sir William Lawrence lived in a style which in our country would be called princely. He was more fortunate or more wise than his renowned cotemporary, Sir Charles Bell, who with all his rare ability died poor and almost broken-hearted. He emulated that other great light of British Surgery, Sir Astley Cooper, who with his great honors accumulated also great wealth. The country seat at which Sir. William spent his evenings was described many years ago by a near relative in a letter from London to the Louisville *Journal.* My kindman was then a young man pursuing his Medical studies abroad, and after speaking of the address and personal appearance of Sir Lawrence, he went on to say: "I found Mr. and Mrs. Lawrence in one of their most splendid green-houses. This was the first country seat I had visited in England, and truly I can say I never set foot on a more lovely spot. Mr. L's entire possessions embrace about fifty acres, and cost him a fraction under $175,000. You approach the house through rows of ancient and full-foliaged elms, whose branches almost sweep the ground. Entering the hall you find yourself surrounded on all sides by statues of Apollo, Diana, the Graces, &c. On your right is the superb library, the entire side of a room fifty feet long covered from the ceiling to the floor with choice and elegantly bound books. Standing in front of the green-house I enjoyed one of the most enchanting views that I ever expect to behold. The conservatory, with its many thousand feet of glass, and flowers filling the air with fragrance; the statues of the Divinities cast in metal or cut in marble; the fountains throwing up their jets of clear water in the same; the stately mansion, the venerable elms, the lake, the grottoes, and the grand old cedars of Lebanon form a picture of surprising beauty and magnificence."

Velpeau died in Paris on the 24th of August. It is an interesting coincidence that he closed his brilliant career while the International Medical Congress was holding its sessions in the Ecole de Medicina. Among the delegates from various parts of the world, there were many of his former pupils in that body who had thus an opportunity of paying their last respects to the remains of their venerated teacher. His funeral was an imposing one. All sections of the Institute of France were largely represented; the National Guard with muffled drums marched in the procession; colleagues, pupils and hundreds of the working people swelled the throng, and the full choral service at St. Thomas d'Aquin was performed in its most impressive style. The testimony of the common people to his worth was affecting. I have seen it stated that a poor woman, on being asked if she knew Velpeau, exclaimed with tears in her eyes: "Yes, upon my word; and he was very good, and very celebrated." His goodness was first in the minds of the poor, who had so often experienced his gentleness and skill at La Charité. Nélaton, who had long been his associate pronounced his eulogium, and gave to the assembled multitude a sketch of his life.

Velpeau was born at Bréche, May 18th, 1795, and was at the time of his death 72 years old. The son of a farrier, or blacksmith, he learned to read in the corner of the forge, in the intervals between shoeing horses. When he had accumulated a little money he went to Tours, where he attracted the attention of Betorneau, and was, through his influence, made an *interne* to the Hospital, then filled with wounded soldiers from Napoleon's battle-fields. In 1820 he made his way to Paris. He won a prize the year following and was made demonstrator of anatomy. In 1823 he was made Doctor of Medicine, and by *coucours* became Surgeon-in-Chief to La Pètié in 1830. In 1824 he succeeded to the chair just made vacant in the Institute of France by the death of the immortal Baron Larrey. Like Ambrose Paré, he rose from the humblest ranks to be a power in France, without ever once leaving his profession to mingle in politics. Of all the Surgeons of whom France can boast he was the most familiar with the medical literature of other countries. And well did his colleague say, addressing his inanimate remains:—"Velpeau, you have a great name. You have taught a grand lesson—that by unceasing work, with a high sense of duty, a man may, without help, in spite of privations and difficulties, achieve true greatness."

It would be interesting to compare and contest the characters of the great surgeons of France and England, who within so short a time of each other have been summoned from their earthly fields of labor; but I must not occupy your space with more than a few words on the subject. In aspect and manner, few men were more unlike than Lawrence and Velpeau. Sir William belonged to the Old School of gentlemen. Velpeau retained through life the impress of his birth and origin, and always resembled a country farmer. But if his head and face gave less promise than the imposing physiognomy of the Englishman, he certainly used his brain to greater advantage. He could never have written the "Natural History of Man," but his rival has left behind him no work equal to Velpeau's great Operative Surgery. With far more genius and better opportunities in youth, Lawrence accomplished less than Velpeau, because he lacked the earnestness, the undivided zeal, the indomitable industry of the "farrier of France." With Velpeau's inflexible will and singleness of aim, there is not a point in the profession to which he might not have risen, and with all John Hunter's powers—incomparably more than his learning—he might to-day have ranked first of English surgeons. The lesson is instructive. But I must pass to more practical subjects, and so bring my letter to a close. Our countryman, Dr. Marion Sims, has won a great reputation in Europe; he is now one of the medical celebrities of this great medical emporium. He has made a decided improvement in the *speculum*, and now uses only the one of his own invention. He prefers light entering his room from a single window directly at his back, and insists on the evacuation of the rectum previously to introducing the instrument. A table four feet long and covered with a blanket is better than a bed or couch. Instead of the back, he has his patients to lie on the left side, diagonally to the table; spine straight with the head, which may be slightly raised, but not the shoulders; chest prone on the table; arms wide apart and not under the body. The thighs are to be bent apart at right angles to the body, and legs at a similar angle, an assistant raising the feet slightly. A small table, a little higher than the other, is convenient for this purpose. In this position, the epigastrium is lower than the pelvis, and the waist being unncompressed, the abdominal viscera gravitate forwards and downwards, and allow air to enter the vagina with the speculum. Dr. Sims ascertains the size, position, and direction of the womb, the patient lying on her back. The left under finger is introduced, while palpation is used with the right hand, pressing down the uterus. Before introducing tent, syringe or knife into the womb, he fixes the cervix with a tenaculum, after introducing the speculum, by which he can draw the organ down and see into the cavity. He calls his instrument the *duck-bill speculum.*

*Of Cholera.*—While it must be confessed that we have not made as great advances in the cure of cholera as might have been hoped from the amount of professional talent bestowed upon the subject, there can be no doubt that much has been achieved in the way of prevention. No more signal service, in fact, has of late years been rendered by our profession to humanity than the tracking of this disease from point to point, and thus determining the mode by which it may be arrested and averted. I see it stated in late journals that the Paunjaub has just been saved from a visitation of the epidemic. Five years ago the troops and prisoners there were decimated by the pestilence. Among the sanitary precautions used were preventing large gatherings of people, and sending troops and convicts into camps. I well remember the success with which a similar practice was pursued on the plantations of Rutherford county, Tennessee, when cholera prevailed there in 1849. So soon as a case of the disease showed itself in a family, the houses were deserted, and masters and slaves betook themselves to tents in the forests, and hardly ever was there any renewal of the epidemic. There can be no doubt that safety in cholera consists in flight from the centres of the poison, or by sanitary measures, preventing its evolution.

*Odd Members.*—Sir James Y. Simpson stated at a late meeting of the Obstetrical Society of Edinburgh, that he had seen in his practice in that city seven cases of intra-uterine amputation, all of the left arm, with rudimentary fingers existing; and he remarked that it was almost universally the left arm that was missing in these cases of odd members. Some years ago Prof. Simpson calculated that if this deformity occurred as often elsewhere as in Edinburgh, which was probably the fact, there must be from forty to fifty thousand such left-handed individuals in the world. The fact of the embryo generally lying on the left side might account for the left arm amputations. He related the case of a girl who had neither arms nor legs, residing in the Highlands of Scotland, and also that of a graduate of the University of Edinburgh, without arms, who wrote his exercises with his feet. In Antwerp, a few weeks ogo, I saw a man without arms, who had made himself a painter, holding his brush in his toes. His touch was wonderfully delicate and his accuracy extraordinary. I am not sure whether he was born without arms or had lost them from injury.

*Anæsthetics.*—In a few places in the world there is still a contest between ether and chloroform as anæsthetics.—Nearly everywhere ether has been thrown into the shade by its rival, but in Lyons, as in Boston, it has held no ground as the safer of the two. A death from ether, however, having taken place in Lyons this summer, a discussion arose on the facts of the case at the Academy of Medicine, which will be likely to rouse public judgment on the subject. A woman of a delicate Constitution inhaled ether to allow of the application of an orthopedic apparatus for some deformity of the foot, and died under its influence. In the discussion to which the case gave rise, it appeared that since the profession of Lyons had resolved to adhere to ether, no less than seven deaths had resulted from its administration, while in Paris, in the nineteen years during which chloroform has been in use, over a much wider range of cases, the casualties have been no greater. The use of anæsthetics seems to be more universal in Europe than in our country, and professional opinion leans more and more to the propriety of their administration. The danger from chloroform is regarded as trifling, provided it is not given too freely at once, but the patient is brought very gradually under its influence.

